# The association between Toll-like Receptor 4, CD14 and coronary atherosclerosis.

**DOI:** 10.1186/1749-8090-10-S1-A273

**Published:** 2015-12-16

**Authors:** Maria Kalliopi Konstantinidou, Nikos Goutas, Nikoleta Poumpouridou, Dimitrios Vlachodimitropoulos

**Affiliations:** 1 Department of Cardiothoracic Surgery, Royal Brompton Hospital, London, UK; 2 Department of Forensic Medicine and Toxicology, University of Athens, Athens, Greece

## Background/Introduction

Immune and inflammatory mechanisms are considered to play a key role in the pathogenesis of atherosclerosis. Toll-like-Receptor4 and CD14 receptors are involved in the intracellular signalling pathway of the innate immune and inflammatory responses against pathogens. Functional polymorphisms of TLR4 and CD14 have so far conflicting impact on coronary artery disease.

## Aims/Objectives

We study the most frequent functional polymorphisms of TLR4 -Asp299Gly, Thr399Ile- and of the promoter of CD14 (T/C-159) aiming to identify if they predispose to coronary artery disease.

## Method

We included two study groups. The study group 1 was consisted of 100 human subjects whose post mortem autopsy revealed severe atheromatosis of their coronary arteries. 100 patients who underwent cardiac Multi-Detector-row-Computed-Tomography (MDCT) and they were found positive for severe coronary atheromatosis were included in the study group 2. Our case group consists of both group 1 and 2. The control group consists of 100 healthy individuals. DNA was obtained from 100 paraffin embedded human aortic tissues and 100 blood samples for our study groups respectively. DNA from the control group was also obtained from blood samples. Genotyping was performed by allele specific PCR or PCR-RFLP analysis.

## Results

There was no statistical difference between case group and control group with regards to the frequencies of 299Gly and 399Ile allele of TLR4. Frequencies of T allele of the CD14 promoter, regarding the of CD14(T/C-159) functional polymorphism, are significantly higher in the control group compared to the case group (37% vs 23.75%), p = 0.05.

## Discussion/Conclusion

The results of this study suggest that functional polymorphisms of TLR4 do not have an impact on coronary atheromatosis for the population we studied. However, polymorphisms of CD14 seem to have a protective role against coronary artery disease. Our next step would be to include more patients in our study and find an easily quantified expression of CD14 that could correlate with coronary atheromatosis.

